# Excessive Folic Acid Mimics Folate Deficiency in Human Lymphocytes

**DOI:** 10.3390/cimb44040097

**Published:** 2022-03-23

**Authors:** Khadijah I. Alnabbat, Ali M. Fardous, Diane C. Cabelof, Ahmad R. Heydari

**Affiliations:** 1 Department of Nutrition and Food Science, Wayne State University, Detroit, MI 48202, USA; kalnabbat@kfu.edu.sa (K.I.A.); ali.fardous@wayne.edu (A.M.F.); diane.cress@wayne.edu (D.C.C.); 2 Department of Food and Nutrition Sciences, King Faisal University, Al Hufūf 31982, Saudi Arabia; 3 Barbara Ann Karmanos Cancer Institute, Wayne State University, Detroit, MI 48202, USA

**Keywords:** folate, folic acid, restriction, depletion, excess, supraphysiological, UMFA, LCL, lymphocyte

## Abstract

Food fortification with synthetic folic acid (FA), along with supplementation, results in a marked increase in the population total of serum folates and unmetabolized folic acid (UMFA). Despite the success in reducing neural tube defects at birth in the intended target population (women of childbearing age), the potential deleterious effects of chronically high levels of UMFA in susceptible segments of the population require further investigation. In this study, we examine the effects of FA concentrations, ranging from depletion to supraphysiological levels, on markers of proliferation, DNA methylation, and DNA damage and repair in a human lymphoblastoid cell line (LCL). We note that both low and high levels of FA similarly impact global DNA methylation, cytome biomarkers measured through the CBMN assay, DNA damage induced by oxidative stress, and DNA base excision repair gene expression.

## 1. Introduction

An optimal intake of dietary folate is essential as mammalian cells lack the enzyme required for folate biosynthesis [1,2]. Folate plays a crucial role in cell proliferation, DNA repair, energy metabolism, amino acid metabolism, and neurotransmitter synthesis. These functions of folate are mediated by the biologically active form tetrahydrofolate (THF) and its derivatives [3]. Folate deficiency is implicated in the pathogenesis of various diseases, including cancer, cardiovascular disease, neurocognitive disorders, anemias, and many more [4]. Folate is crucial during the reproductive lifecycle, and low folate status is associated with neural tube defects (NTDs), leading several countries, including the USA, Canada, and Chile, to implement mandatory fortification programs of food with folic acid (FA) in bread, cereal, and grain products [5]. This fortification has led to a significant (19–32%) reduction in NTDs [6,7] and a corresponding increase in the population total of serum folate [8]. Since then, many in the scientific community have raised concerns about the safety and unintended consequences of exposing entire populations to high levels of synthetic folic acid [4,9]. 

While folic acid was previously considered safe, with little or no known systemic toxicities, emerging evidence reveals that chronically elevated levels of FA are associated with deleterious effects [4,10]. Some have suggested that synthetic folates, principally folic acid, can lead to deleterious effects compared to natural folates [11,12]. Because of their reduced stability and bioavailability, consumption of natural folates from the diet is unlikely to lead to excessively elevated levels, while on the other hand, FA is highly stable and bioavailable [13]. Emerging in the center of this controversy is the potential role of unmetabolized folic acid (UMFA) in the etiology of the observed deleterious effects [12,14,15]. Unlike natural folate, FA must be activated through a two-step reduction by dihydrofolate reductase (DHFR) [16] before participating in the one carbon cycle, followed by conversion to 5-Methyltetrahydrofolate (5-mTHF) by methylenetetrahydrofolate reductase (MTHFR) in a B2-dependent reaction [3]. The presence and persistence of UMFA in the blood are thought to be a result of low and variable DHFR activity in humans, as well as the inhibitory effect of FA on DHFR, which leads to saturation of the biotransformation of FA [17,18]. Following FA fortification, UMFA was detected in the majority of sampled U.S. children, adolescents, and adults [19,20,21,22]. The significance of these observations stems from the potential of UMFA to impair folate metabolism by inhibiting DHFR and MTHFR [23,24]. While the potential consequences of UMFA disrupting folate metabolism and the one-carbon cycle are still controversial, recent reports have suggested that excessive FA supplementation can mimic folate insufficiency in model organisms [25,26,27,28]. A study of *C. elegans* revealed that high doses of FA induce severe oxidative stress and an accumulation of homocysteine (Hcy) [28], while others showed that both folate deficiency and excess impair folate metabolism [27]. In a mouse model, Henry et al. found that both dietary folate deficiency and FA supplementation similarly impair folate-dependent biosynthetic pathways in lymphocytes [26]. 

In this study, we tested whether excessive folic acid exposure to a human lymphoblastoid cell line can mimic folate restriction and induce functional folate deficiency in vitro. Folate deficiency leads to uracil misincorporation into DNA, generating point mutations, single- and double-strand breaks, and chromosome breakage [29,30,31]. Folate (5-mTHF) is also required for the conversion of homocysteine to methionine in a B12-dependent reaction. Therefore, a decrease in methionine regeneration leads to a decline in S-adenosyl methionine (SAM) synthesis, a methyl donor essential for several cellular and DNA methylation reactions, which in turn leads to alterations in DNA methylation patterns and gene expression, and eventually chromosomal aberrations [29,32]. To assess the impact of folic acid levels on LCLs, we used folate-free media supplemented with FA in a range of concentrations adapted from previously published reports, where 12 nM represents the depletion level [29], 180 and 300 nM represent proposed optimal levels [29,33,34], 2300 nM represents the routine cell culture media supplementation level, and 10,000 nM represents the supraphysiological level [35,36,37]. We then assessed the impact of FA supplementation on proliferation, homocysteine levels, global DNA methylation, and DNA damage and repair. 

## 2. Materials and Methods

### 2.1. Cell Culture

The human lymphoblastoid cell line (GM16113) (Coriell institute, Camden, NJ, USA), was cultured in a folate-free RPMI-1640 medium (Sigma-Aldrich, St. Louis, MO, USA), supplemented with 10% dialyzed FBS, 1% penicillin/streptomycin (5000 IU penicillin/5 mg streptomycin), 1% glutamax, and 1% sodium pyruvate, and incubated in a humidified atmosphere with 5% CO_2_ at 37 °C. Cells were initially grown in a medium containing a final concentration of 300 nM FA for 3 passages before seeding cells in either 12, 180, 300, 2300, or 10,000 nM FA.

### 2.2. Doubling Time

Cells were seeded at the selected concentrations in a 12-well culture plate, and counted after 4 h, as T = 0, and then after 24, 48, and 72 h using trypan blue and an automated cell counter, TC20 TM (Bio-Rad, Hercules, CA, USA). The cell population doubling time (DT) is reported as the mean of 3 days and was calculated using the formula: DT = In2/In (Xe/Xb), where T is the incubation time in any units, Xb is the cell number at the beginning of incubation time, and Xe is the cell number at the end of the incubation time. 

### 2.3. Homocysteine Assay

Cells were washed twice with ice-cold 1× PBS and then collected by centrifugation at 2000× *g* for 10 min at 4 °C. The cell pellet was homogenized on ice in 1 mL ice-cold 1× PBS, then centrifuged at 10,000× *g* for 15 min at 4 °C. The supernatant was removed and stored on ice. The homocysteine level was determined using a commercially available Homocysteine ELISA kit (Cell Biolabs, San Diego, CA, USA), according to the manufacturer’s instructions.

### 2.4. LINE-1 Methylation Assay

Genomic DNA was isolated using a PureLink^®^ Genomic DNA Mini Kit (Life Technologies, Carlsbad, CA, USA), following the manufacture protocols. LINE-1 methylation assay was performed using a Global DNA Methylation LINE-1 kit (Active Motif, Carlsbad, CA, USA). Briefly, 100 ng Msel-digested genomic DNA was hybridized to the LINE-1 probe and immobilized onto a streptavidin-coated plate. After the binding of primary and secondary antibodies, data were obtained and analyzed through a colorimetric plate reader reaction by comparison to a standard curve of methylated and nonmethylated DNA.

### 2.5. Cytokinesis-Block Micronucleus (CBMN) Assay

This assay was adopted from protocols published by Thomas and Fenech [38]. Cells were initially grown in a folic-acid-supplemented medium at 300 nM. Cells were washed twice in Hank’s balanced saline solution (HBSS), then incubated in a supplemented RPMI-1640 medium containing either 12, 180, 300, 2300, or 10,000 nM of folic acid and at a final concentration of 4.5 μg/mL cytochalasin B (Sigma Aldrich, St Louis, MO, USA). After 24 h, cells were harvested in duplicate using a Shandon Cytospin 4 (Thermo Scientific, Waltham, MA, USA) at 600 rpm for 5 min. Slides were air-dried, fixed, and stained using Shandon Kwik-Diff Stains (Thermo Scientific, Waltham, MA, USA), and cover-slipped using DPX mountant (Sigma Aldrich, St. Louis, MO, USA). The frequency of MNi, NPBs, and NBUDs (cytome biomarkers) was determined in 2000 binucleated (BN) cells, following the scoring criteria of HUMN project guidelines [39]. Slides were coded and scored by two trained scorers in a blinded manner. Cytome biomarker scores are presented per 1000 binucleated (BN) cells. A schematic of the CBMN assay is presented in Supplemental Figure S1.

For the second part of the experiment involving the exposure of LCLs to hydrogen peroxide and the CBMN assay, we followed the protocols published by Main et al. [40,41]. Briefly, on day 9 of the assay, cells were washed twice in HBSS. Then, cells were exposed to RPMI-1640 medium supplemented with a final concentration of 100 μM hydrogen peroxide for 1 h. The cells were then washed again in HBSS and resuspended in RPMI-1640 cell culture media containing either a 180, 300, or 2300 nM final concentration of folic acid before exposure to cytochalasin B to complete the CBMN assay, as described earlier.

### 2.6. Gene Expression Profiling

Total RNA was extracted from LCLs using TRIzol^®^ Reagent (Gibco BRL, Rockville, MD, USA). First-strand cDNA synthesis was performed using an ImProm-II^TM^ Reverse Transcription System (Promega, Madison, WI, USA). The mRNA expression level of selected genes was assessed with quantitative real-time PCR (qPCR) (PikoReal 96, Thermofisher, Vantaa, Finland), and normalized to the geometric mean of HPRT1 and ß-actin using the 2^−(∆∆Cq)^ method. Primers were validated and tested using external standards for each gene and prepared by subcloning using a TOPO^®^ TA Cloning^®^ kit (Invitrogen, Carlsbad, CA, USA). Primer sequences are provided in Supplemental Table S1.

### 2.7. Statistical Analysis

Statistical significance between means was determined using one-way and two-way ANOVA, followed by Tukey’s post hoc test when appropriate (GraphPad Software V9.3.1, San Diego, CA, USA). Any *p*-values of less than 0.05 were considered statistically significant.

## 3. Results

### 3.1. Doubling Time

**Figure 1:**
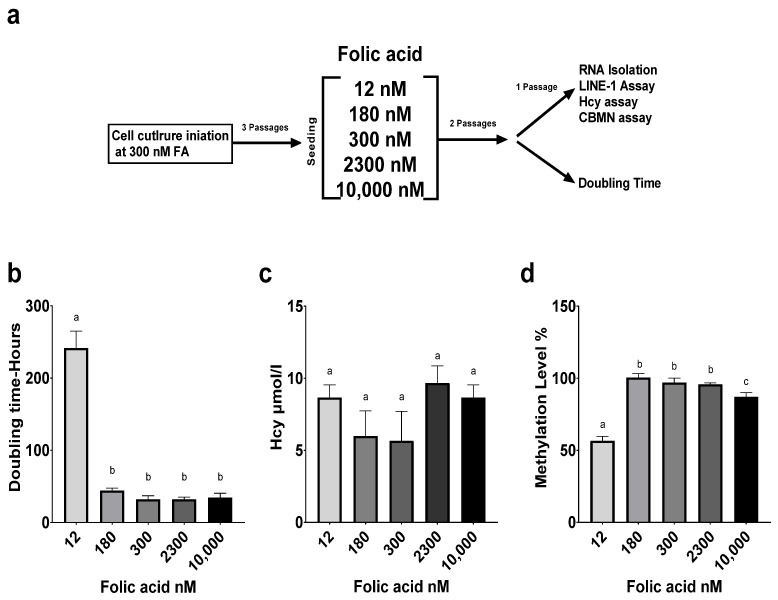
Impact of folate supplementation on proliferation, homocysteine levels, and DNA methylation in human lymphoblastoid cell line. (**a**) Study design. (**b**) Effect of FA media concentration on LCL proliferation. (**c**) Effect of FA media concentration on LCL homocysteine levels. (**d**) Effect of FA media concentration on global DNA methylation through LINE1 Assay. Results are from technical triplicates (*n* = 3). Bars, SEMs, and means with different letters (a,b,c) are significantly different (Tukey’s HSD, *p* < 0.05).

Folate status impacts the proliferation and growth of a variety of cells, including lymphocytes [41,42]. Folate deficiency was shown to lead to nucleotide imbalance, impacting DNA and RNA synthesis and leading to cell cycle arrest and lymphocytopenia [43]. The doubling time (DT) of LCLs typically ranges between 18 and 36 h. Cells grown at 12 nM FA experienced significantly reduced proliferation, as highlighted by an increased doubling time compared to all other FA concentrations (*p* < 0.0001). There was no significant difference in the measured doubling time across all other concentrations (Figure 1b). 

### 3.2. Homocysteine Level

Homocysteine is a well-accepted marker and indicator of folate deficiency [3]. Plasma homocysteine is inversely correlated with serum folate levels in humans [44,45]. Homocysteine levels in our study’s LCLs did not vary significantly across FA concentrations (Figure 1c) 

### 3.3. LINE-1 Methylation Assay

Long interspersed nuclear elements (LINE-1) are mobile parasitic genetic elements that comprise 17% of the human genome, and their methylation levels are considered a surrogate marker of global genomic DNA methylation. Folate depletion has been associated with decreased lymphocyte methylation [45]. Supplementation with 12 nM FA yielded a significant reduction in LINE-1 methylation compared to all other groups (*p* < 0.001). Supraphysiological supplementation at 10,000 nM FA also reduced methylation levels (*p* < 0.05). There was no significant difference in methylation across the 180, 300, and 2300 nM FA concentrations (Figure 1d).

### 3.4. Cytome Biomarker Analysis

**Figure 2:**
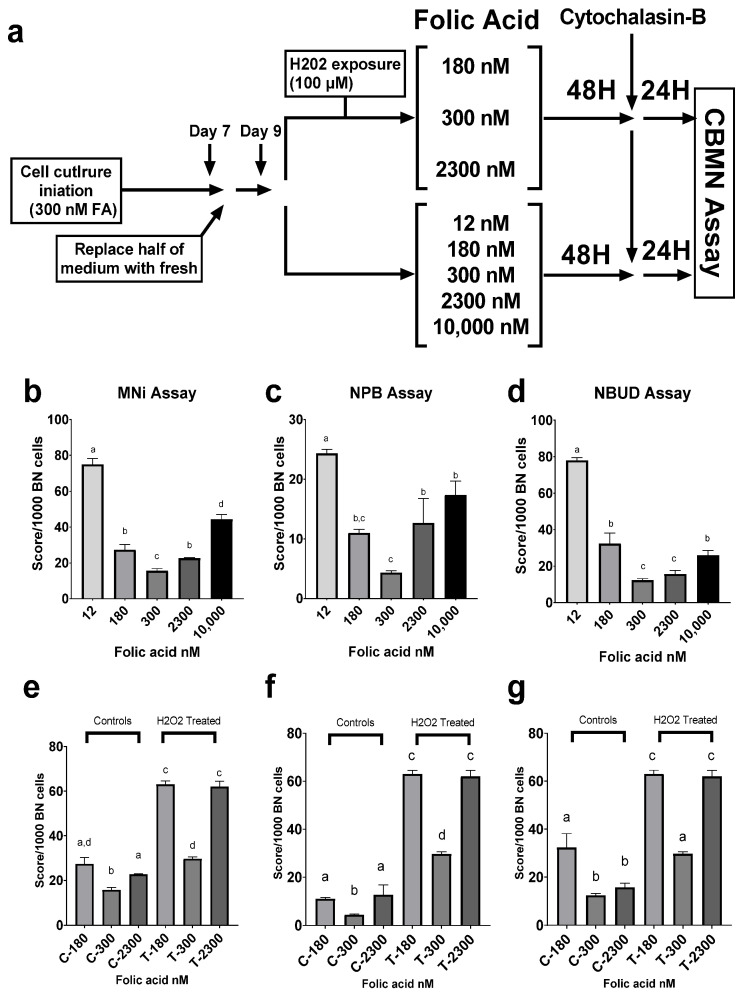
Impact of folate supplementation on markers of genomic damage, measured through CBMN assay in a human lymphoblastoid cell line. (**a**) Study design. (**b**) Effect of FA media concentration on micronuclei occurrence. (**c**) Effect of FA media concentration on the incidence of nucleoplasmic bridges. (**d**) Effect of FA media concentration on nuclear budding. (**e**) Media FA levels impact the incidence of micronuclei in LCLs exposed to hydrogen peroxide. (**f**) Media FA levels impact the incidence of nucleoplasmic bridges in LCLs exposed to hydrogen peroxide. (**g**) Media FA levels impact the incidence of nuclear budding in LCLs exposed to hydrogen peroxide. Results are from technical triplicates (*n* = 3). Bars, SEMs, and means with different letters (a,b,c,d) are significantly different (Tukey’s HSD, *p* < 0.05).

The CBMN assay measures endpoints for DNA damage, such as micronuclei (MNi), which are a biomarker of chromosome breakage and/or whole chromosome loss; nucleoplasmic bridges (NPBs), which are a biomarker of DNA misrepair and/or telomere end-fusions; and nuclear buds (NBUDs), which are a biomarker of elimination of amplified DNA and/or DNA repair complexes. MNi in lymphocytes are very sensitive to small changes in micronutrient status, including folate, which makes them a robust biomarker to identify the impact of folate status on genome stability [46,47,48]. Cells supplemented with 300 nM Fa exhibited the lowest number of cytome biomarkers overall (*p* < 0.05). Supplementation with the lowest level of FA (12 nM), representing a folate-deficient state, resulted in the highest score across the three scoring criteria, indicating extensive genomic damage (*p* < 0.0001). Supraphysiological FA supplementation (10,000 nM) resulted in a significant increase in MNi compared to the midrange FA concentrations (180, 300, and 2300 nM FA, *p* < 0.005), an increase in NPBs, compared to 300 nM FA (*p* < 0.005), and an increase in NBUDs, compared to 300 and 2300 nM FA (*p* < 0.05) (Figure 2b–d). Further testing was performed on the concentrations that were in the optimal range of FA supplementation to assess DNA damage response to oxidative stress upon exposure of LCLs to hydrogen peroxide, compared to untreated controls. The results showed that FA supplementation at 300 nM resulted in the lowest score of cytome biomarkers in both untreated controls and treated cells (*p* < 0.05 and *p* < 0.0001, respectively). 

### 3.5. Gene Expression Analysis

**Figure 3:**
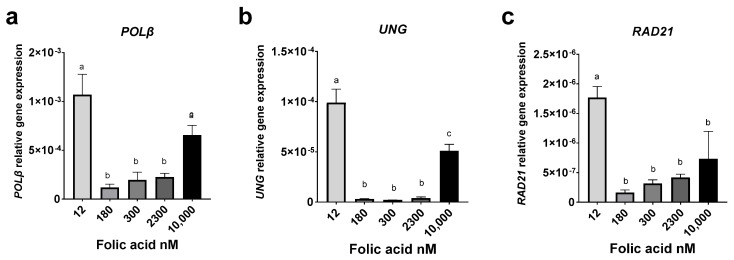
Impact of folate supplementation on the relative gene expression of DNA repair genes. (**a**) Effect of FA media concentration on the relative gene expression of *POLβ*. (**b**) Effect of FA media concentration on the relative gene expression of *UNG*. (**c**) Effect of FA media concentration on the relative gene expression of *RAD21*. Results are from technical triplicates (*n* = 3). Bars, SEMs, and means with different letters (a,b,c) are significantly different (Tukey’s HSD, *p* < 0.05).

Folate deficiency causes uracil misincorporation, resulting in DNA damage and chromosomal breakage [31]. Uracil in DNA is repaired primarily by the base excision pathway (BER) through an initial removal by uracil DNA glycosylase (*UNG*, or *UDG*), followed by a multistep process culminating in the repair of the damaged sites by the rate-limiting enzyme DNA polymerase β (*Polβ*). Our previous work revealed that folate deficiency can impact and regulate the base excision repair pathway [49,50]. Disruption of critical BER enzymes and pathways can lead to single- and double-strand DNA breaks [49,50,51]. *RAD21* is a key gene involved in cellular S-phase arrest and the repair of double-strand breaks by homologous recombination (HR) and sister chromatid cohesion [52]. FA deficiency (12 nM) resulted in the highest relative gene expression of the genes *POLβ*, *UNG*, and *RAD21*, compared to all other levels of FA supplementation (*p* < 0.05) (Figure 3a–c). However, we noted that the 10,000 nM supraphysiological FA concentration resulted in a significant increase in relative gene expression of *POLβ* and *UNG* compared to the 180, 300, and 2300 nM FA concentrations (*p* < 0.05) (Figure 3a,b). 

## 4. Discussion

Lymphocytes are accepted as a sensitive model with which to examine the effects of folate status on genomic stability markers such as strand breakage, microsatellite instability, methylation, and uracil misincorporation [48,53,54,55]. Total lymphocyte folate closely correlates with plasma folate and homocysteine [53]. Moreover, high folate intake and UMFA have been linked to a decreased cytotoxicity of natural killer cells in aged mice and postmenopausal women, respectively [15,56]. Lymphoblastoid cell lines (LCLs) are spontaneously replicating lymphocytes established via in vitro infection with the Epstein–Barr virus to produce a convenient alternative to isolated blood lymphocytes. These cell lines have minimal genetic and phenotypic changes and are considered a suitable model for functional and genetic studies [57].

Folate is crucial for nucleotide synthesis and DNA methylation, and it is involved in multiple pathways that are essential for cellular proliferation and homeostasis. We noted that folate depletion (12 nM FA) resulted in a significant increase in doubling time in our LCLs. However, no significant difference in the cellular proliferation rate was observed across the rest of FA-supplemented cell cultures. Some suggested that excess FA can cause an increase in UMFA, leading to saturation of DHFR and impacting the dynamics of the one carbon cycle [28]. A study by Ortbauer et al. on *C. elegans* revealed more intricate dynamics: both folate deficiency and over-supplementation disrupted folate homeostasis by favoring thymidylate synthase over the methionine synthase cycle [27]. This implies that the folate cycle favors nucleotide synthesis, and therefore DNA maintenance and proliferation, over methylation reactions at both extremes of FA status. Despite the proposed shift in the folate cycle, we did not detect a significant difference in homocysteine levels in our LCLs at any of our FA concentrations. However, a U-shaped trend was observed. While folate depletion commonly causes hyperhomocysteinemia, others showed that excess FA can also lead to a significant accumulation of homocysteine, resulting in severe oxidative stress in *C. elegans* [28]. Although there is an abundance of reports linking folate status to plasma homocysteine, leading to direct effects on lymphocytes, we were unable to locate comparative reports that examined the effects of FA on lymphocyte homocysteine levels in vitro. 

Assessment of DNA methylation through the LINE-1 assay revealed that both FA depletion (12 nM) and supraphysiological FA (10,000 nM) produced a decrease in global methylation compared to 180 nM FA. While folate depletion is known to be linked to global hypomethylation of DNA [58], the link between FA excess and DNA methylation is not well-defined. Charles et al. showed that supraphysiological levels of FA significantly reduce LINE-1 methylation levels in human lung fibroblasts and colon epithelial cell lines, and that the impact is passage-dependent [36]. Hypomethylation of CpG islands leads to heterochromatin defects, centromere instability, and chromosome malsegregation, and eventual loss as MNi [29,59,60]. A Brazilian cross-sectional study of a population exposed to mandatory FA fortification by Steluti et al. did not find an association between global DNA methylation and folate status. A study comprising a cohort of pregnant Canadian women revealed that serum, RBC folate, and UMFA were not significantly associated with fetal DNA methylation, but an inverse correlation with DNA hydroxymethylation was found. Furthermore, rodent studies have linked high FA maternal intake to adverse metabolic and behavioral perturbations in offspring, suggesting that alterations in methylation and inheritable epigenetic changes can be precipitated by FA supplementation [25,61,62,63,64]. Similarly, studies have found a link between high folate status in Indian mothers and insulin resistance and adiposity in children [65,66]. The hypomethylation effect induced by a supraphysiological level of FA can be explained by the limited capacity of cellular DHFR and MTHFR to reduce FA to 5-mTHF, which is the active form required for methionine regeneration and SAM synthesis. Christensen et al., in a mouse model, reported that FA supplementation led to significant declines in mRNA expression and protein activity of MTHFR. The decline in MTHFR activity reduced effective 5-mTHF concentrations in high FA-fed mice compared to controls [67]. A recent report showed that FA supplementation in pregnant mice downregulated MTHFR and altered choline/methyl metabolism in both mother and progeny [25]. It has also been reported that FA supplementation led to the inhibition of MTHFR in crude brain extract [68], as well as in crude liver extract [67], suggesting that UMFA may lead to MTHFR deficiency. The effect of excess folate on global and gene-specific methylation, as well as on epigenetics, represents an important research venue to address the safety of FA supplementation in human populations. 

Folate deficiency is known to induce genomic instability due in part to a decrease in thymidylate synthesis and uracil misincorporation in DNA, leading to single- and double-strand breaks, impaired repair, and genomic damage. The CBMN assay, utilizing the micronucleus index (MNi), is one of the standard methods of cytogenetic and genetic toxicology testing [69]. The MNi is derived from events that lead to chromosome fragments or whole chromosomes to lag during anaphase throughout nuclear division [59]. As expected, FA deficiency results in a significant increase in all cytome biomarkers; however, we observed a significant increase in MNi scores, as well as an upward trend in NPBs and NBUDs at 10,000 nM compared to 180, 300, 2300 nM FA, (Figure 2b–d). This implies that FA excess mimicked folate depletion’s effects on DNA damage and repair, but the underlying mechanisms require further investigation. It is inviting to suggest that at 10,000 nM FA, DNA hypomethylation along with aberrant DNA repair capacity, when combined with a normal proliferative response, can amplify the deleterious effects, while under FA depletion (12 nM), proliferation was significantly inhibited. Analysis of the base excision repair genes, *UNG* and *POLβ*, revealed a similar trend where FA excess mimicked the effect of FA depletion (Figure 3a,b), and revealed a recurring U-shaped relationship between deleterious effects and ranges at either extreme of FA supplementation. Taken together, we speculate that the increase in MNi incidence under excess FA may be linked to genomic hypomethylation and uracil misincorporation events. The link between uracil incorporation into DNA and consequent generation of double-strand breaks (DSB) is well-established [49]. Constant removal of uracil by *UNG*, followed by incomplete repair of generated gaps by *POLβ*, leads to DSBs, chromosome breakage, and chromosome loss [50,70]. These chromosomes are eventually eliminated from the nucleus as MNi expressed in the cytoplasm [59,69,71]. Except for a significant increase at 12 nM, *RAD21* gene expression did not vary significantly across the remaining FA concentrations (Figure 3c), though a familiar U-shaped trend was observed. Similarly, Henry et al. showed that both a supraphysiological FA diet (10 mg/kg) and an FA-deficient diet (0.1 mg/kg), maintained for 5 months, had a comparable impact on mice, leading to compromised nucleotide metabolism, pyrimidine metabolism, low lymphocyte numbers, and impaired B-cell hematopoiesis [26]. 

Our analysis revealed that FA supplementation can differentially impact how LCLs react to genotoxic stress within the optimal range of FA concentration. These concentrations (180, 300, and 2300 nM) are representative of proposed optimal culture conditions. Exposure to hydrogen peroxide revealed that cells incubated in 300 nM FA media experienced the least damage (Figure 2e–g). This finding implies that this concentration may be optimal for this specific cell line, but the results cannot be generalized to other cell lines or extrapolated to in vivo conditions in humans. The presence of diverse genotypes among the human population and the prevalence of polymorphisms that can alter an individual’s folate metabolism and DNA repair capacity, create challenges for researchers. It is important to note that because of such variances, excess FA may not provide benefits to many impacted by the fortification mandate. Although FA fortification has undeniable benefits, additional research is needed to understand the potential deleterious effects of UMFA, thereby allowing us to carefully reassess the landscape ahead.

In summary, our findings corroborate what others have uncovered in various models. Deficient and excessive levels of FA similarly impact global DNA methylation, cytome biomarkers, and DNA repair gene expression. While the specific mechanism underlying the observed effects requires additional research, the accumulation of evidence from recent reports warrants a fresh perspective on the role of folic acid fortification in health and disease.

## Data Availability

The data that supports the findings of this study are available from the corresponding author (A.R.H.), upon reasonable request.

## Supplementary Materials

The following supporting information can be downloaded at: https://www.mdpi.com/article/10.3390/cimb44040097/s1, Supplemental Figure S1: CBMN assay schematic, Supplementary Table S1: Qiagen-RT^2^ qPCR Primers.
