# Supplementary material for: Excessive Folic Acid Mimics Folate Deficiency in Human Lymphocytes
Source: Curr Issues Mol Biol. 2022 Mar 23;44(4):1452–62. doi: 10.3390/cimb44040097 (PMC9164024; doi:10.3390/cimb44040097)
Supplement: Supplementary file 1 [file cimb-44-00097-s001.zip › cimb-1630012-supplementary.pdf]

current issues in molecular biology

MDPI

Excessive Folic Acid Mimics Folate Deficiency in Human Lymphocytes

Khadijah I. Alnabbat [1,2], Ali M. Fardous [1], Diane C. Cabelof [1] and Ahmad R. Heydari [1,3,*]

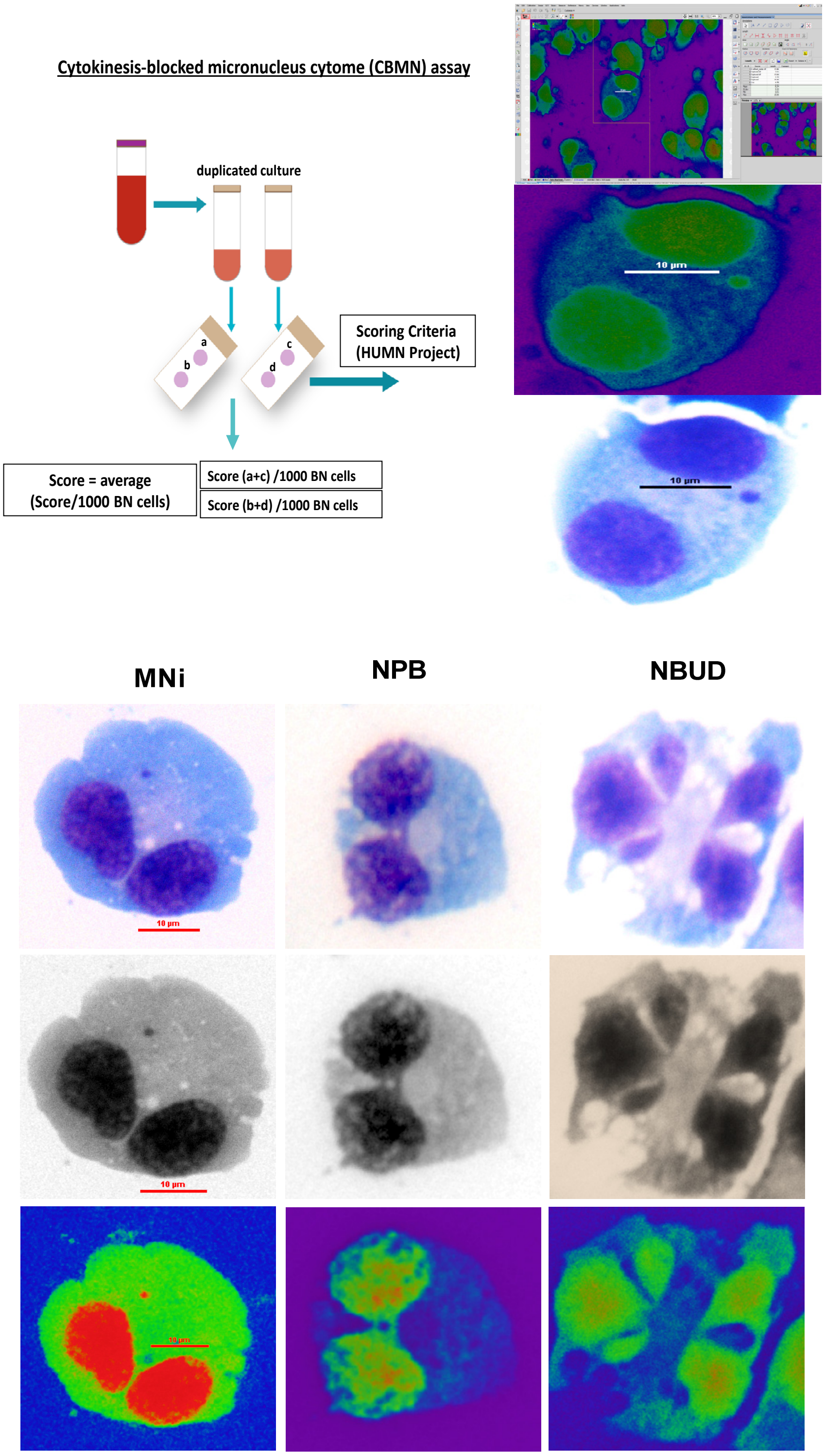

Supplementary Figure S1. CBMN assay schematic.

Supplementary Table S1. Qiagen-RT^2 qPCR Primers.

Gene Symbol	Gene name	NM_Number	Band Size	Reference position	Catalog number
HPRT1	Hypoxanthine phosphoribosyl transferase 1	NM_000194	57	332	PPH01018C-200
ACTB	Beta Actin	NM_001101	174	730	PPH00073G-200
UNG	Uracil-DNA glycosylase	NM_003362	84	2068	PPH01727E-200
POLB	DNA Polymerase Beta	NM_002690	112	139	PPH13735F-200
RAD21	Human RAD21 cohesion complex	NM_006265	142	2124	PPH10216A-200
